# An Unusual Hybrid Salivary Gland Tumor: Molecular Analysis Informs the Potential Pathogenesis of This Rare Neoplasm

**DOI:** 10.1155/2019/2713234

**Published:** 2019-03-28

**Authors:** Yan Zhou, Ernesto Martinez Duarte, David J. Eleff, Laura J. Tafe, Jason M. Leibowitz, Darcy A. Kerr

**Affiliations:** ^1^Department of Pathology and Laboratory Medicine, University of Miami/Jackson Memorial Hospital and Miller School of Medicine, Miami, FL, USA; ^2^Department of Otolaryngology Division of Head and Neck Surgery, University of Miami/Jackson Memorial Hospital and Miller School of Medicine, Miami, FL, USA; ^3^Department of Pathology and Laboratory Medicine, Dartmouth-Hitchcock Medical Center and Geisel School of Medicine at Dartmouth, Lebanon, NH, USA

## Abstract

**Background:**

The presence of two or more tumor entities growing in adjacent locations within the salivary gland is very rare, and pathologic studies on such lesions are limited, particularly those with molecular information. Since the clinical history and imaging studies are usually nonspecific, accurate diagnosis and clinical management largely depend on a thorough histological examination.

**Methods and Results:**

We describe a 71-year-old man with an unusual case of hybrid salivary gland tumor composed of mucoepidermoid carcinoma and basal cell adenoma. Molecular analysis revealed differing driver genetic alterations in each component.

**Conclusions:**

Hybrid salivary gland tumors are rare, and their pathogenesis is controversial. The combination of mucoepidermoid carcinoma and basal cell adenoma has not been previously described. While malignant transformation of adenoma to carcinoma seems plausible, gene sequencing was more suggestive of their independent derivation. Key to appropriate surgical management is identifying the more aggressive component, ideally at the time of intraoperative consultation.

## 1. Introduction

In salivary gland, the presence of two or more histologically distinct tumor entities in one topographic location is very rare [1]. Here we encountered a case in which a patient presented with a single parotid mass consisting of two histologically distinct tumors: mucoepidermoid carcinoma (MEC) and basal cell adenoma (BCA), membranous type. The juxtaposition of distinct tumor types raises various pathophysiologic possibilities; namely, did the tumors arise independently or did one tumor type arise from the other? Using clinicopathologic and molecular evaluation of the two tumor components, we believe the current case represents an unusual hybrid salivary gland tumor. Herein, we discuss the findings, differential diagnoses and clinical implications raised by this unique case.

Hybrid salivary gland tumors are very rare, with an incidence of less than 0.1% of all salivary gland tumors [2]. The entity is defined as “a neoplasm composed of two or more separate different tumor entities, each one of which conforms to an exactly defined tumor category, arising within the same topographical area” [1–6]. While most reported cases are composed of exclusively two or more malignant entities, the tumor components can be either benign or malignant [2, 6]. The most frequently found components in hybrid salivary gland tumors are adenoid cystic carcinoma, epithelial-myoepithelial carcinoma, and salivary duct carcinoma, with adenoid cystic carcinoma commonly being the predominant component [6, 7]. Both mucoepidermoid carcinoma and basal cell adenoma have been described as possible tumor components [2, 7, 8]. However, the combination of mucoepidermoid carcinoma and basal cell adenoma in a single tumor has not been reported yet to our knowledge. Due to their rarity and heterogeneous nature, prognostic evaluation and clinical management cannot be standardized for hybrid salivary gland tumors. In practice, the management is often guided by the most aggressive type within the hybrid tumor which may represent a minor component of the overall lesion [6]. Therefore, thorough histologic sampling of salivary gland tumors is critical to the recognition of hybrid tumor and may drive clinical management.

## 2. Case Report

### 2.1. Clinical Findings

A 71-year-old man with a 25 pack-year history of tobacco use presented for continuing care, and physical examination revealed a painless right parotid gland mass previously unnoted by the patient. It was palpable as a soft 0.5 cm mass. Cranial nerve examination was without deficits, and no cervical lymphadenopathy was detected. He had a history of left parotidectomy for Warthin tumor three months prior and Mohs surgery of the right cheek for nonmelanoma skin cancer five years prior. CT scan revealed a 1.4 x 1.3 cm right superficial parotid mass. For diagnostic and therapeutic purposes, a right superficial parotidectomy was performed. Intraoperative frozen section examination revealed mucoepidermoid carcinoma, intermediate grade. Thus, the decision was made to perform a right deep lobe parotidectomy with preservation of facial nerve and right neck dissection. The patient underwent definitive IMRT 60Gy radiation therapy following recovery from the surgery and has been followed up for four months.

### 2.2. Pathological Findings

**Figure 1:**
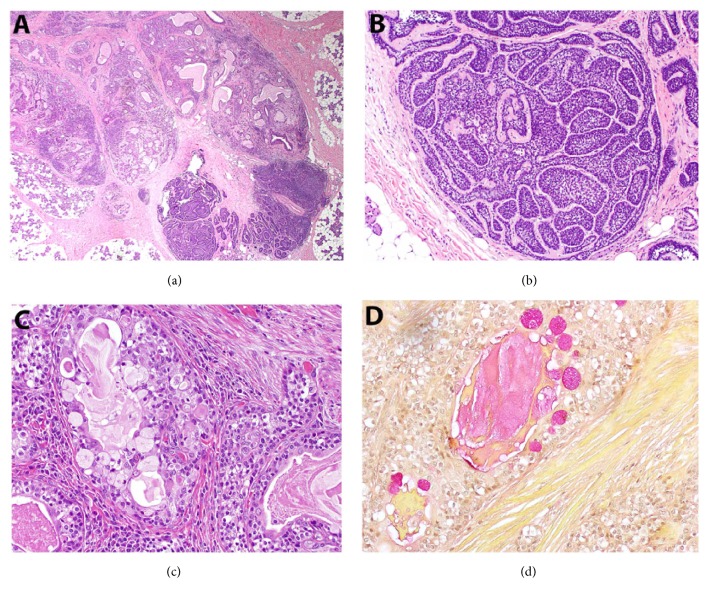
Histopathological findings of the parotid gland mass. (a) A low-power view shows two morphologically distinct lesions immediately adjacent to each other: mucoepidermoid carcinoma (upper left) and basal cell adenoma (lower right) with a sharp transition between the two components. (b) The basal cell adenoma component demonstrates jigsaw-like nests of basaloid cells surrounded by a ribbon-like rim of eosinophilic hyaline material. (c) The mucoepidermoid carcinoma component shows numerous plump mucocytes with finely granular clear cytoplasm as well as polygonal epidermoid cells and intermediate cells. (d) A mucicarmine special stain confirms the presence of intracytoplasmic and extracellular mucin associated with the lesional cells of the mucoepidermoid carcinoma.

**Figure 2:**
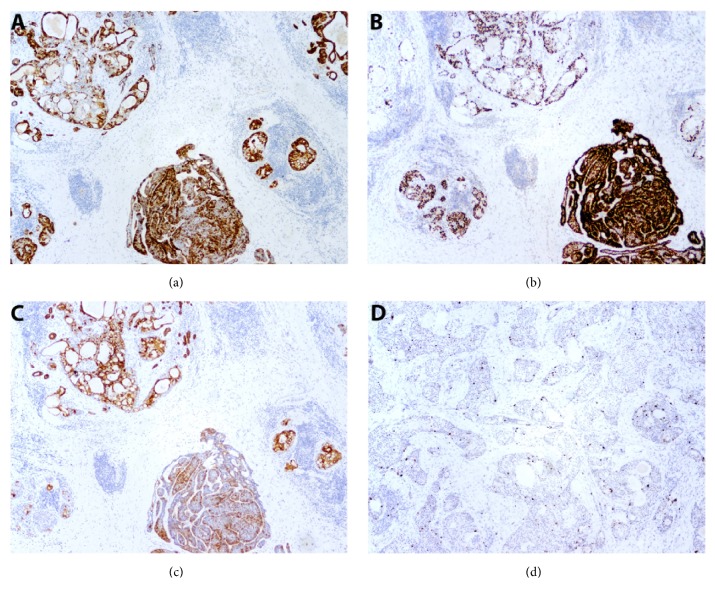
Immunohistochemical findings of the parotid gland mass. (a) A CK5/6 stain highlights the peripheral abluminal squamoid cells in mucoepidermoid carcinoma (upper left) as well as a prominent abluminal myoepithelial cell population in the basal cell adenoma (lower middle). (b) Similarly, p63 shows peripheral positivity in both tumor components. (c) A stain for CK7 highlights mucocytes and cells adjacent to glandular lumens in the mucoepidermoid carcinoma and patchy cells throughout the basal cell adenoma with a subset of luminal cells staining intensely. (d) The proliferation index as estimated by an immunohistochemical stain for Ki-67 is approximately 3-4% in the mucoepidermoid carcinoma; the basal cell adenoma is no longer present in the deeper level used for immunohistochemistry.

On gross examination of the resected right superficial parotidectomy specimen, serial sections revealed a 1.3 x 1.1 cm firm, tan-white intraparenchymal tumor nodule with ill-defined borders. In addition, a cyst measuring 0.6 cm was present 1.1 cm away from this tumor, with grossly unremarkable intervening parenchyma. Histologic examination of the nodule showed two distinct lesional components that were well-demarcated from each other with no transition zone (Figure 1(a)). One portion showed well-circumscribed multinodular proliferation composed of dark blue, basaloid tumor cells arranged in nests with frequent peripheral palisading. Cytologically, the tumor cells demonstrated ovoid, basophilic nuclei and scant cytoplasm. Distinct, dense ribbons of eosinophilic hyaline material were noted surrounding the islands of tumor cells (Figure 1(b)). These histologic findings were those of basal cell adenoma, membranous type. Immunohistochemical stains for p63 and CK5/6 highlighted a prominent abluminal population, and CK7 highlighted patchy cells throughout, with a subset of luminal cells staining intensely (Figure 2). The other portion of the tumor was characterized by a multinodular proliferation of multiple distinct cell populations arranged in frequent mucin-containing glandular spaces and as solid tumor cell nests. The lesional cells consisted of an admixture of mucous cells (mucocytes), polygonal epidermoid cells, and intermediate cells (Figure 1(c)). A special stain for mucicarmine highlighted intracellular mucin within the mucocytes as well as the extracellular mucin (Figure 1(d)). Cytologic atypia was moderate to focally marked. Necrosis was not seen and the mitotic activity was scant. Immunohistochemical stains for p63 and CK5/6 highlighted a predominant abluminal epidermoid/squamoid cell population, and CK7 highlighted mucocytes and cells adjacent to extracellular mucin of the glandular lumens. The Ki-67 proliferation index was approximately 3-4% (Figure 2). The histologic findings were consistent with intermediate-grade mucoepidermoid carcinoma. Microscopically, the grossly noted 0.6 cm cyst consisted of a simple cyst composed of cytologically bland cuboidal lining cells with focal mucinous metaplasia, consistent with a salivary duct cyst. The parenchyma between this region and the tumor was histologically unremarkable as was the deep lobe of the parotid. Eighteen sampled lymph nodes were negative for tumor.

To further investigate the association between the two tumor components, molecular profiles of both were analyzed by next-generation sequencing (NGS) (FoundationOne®, Cambridge, MA). Among the 315 genes tested, the MEC and BCA components shared five genomic variants in common, all characterized as variants of unknown significance (VUS). Additionally, each showed one or two distinct likely driver genomic alterations (Table 1). Interphase fluorescence in situ hybridization (FISH) for* MAML2* rearrangement, a genetic alteration commonly found in MEC, was attempted, but testing was uninformative as no interpretable hybridization signals were present to complete the analysis.

## 3. Discussion 

There are only a few articles describing two or more neoplastic entities growing in adjacent locations within the salivary gland. Genetic analyses of the tumor components that may provide useful information regarding tumorigenesis are even more limited [4, 9]. When encountering such cases, several important differential diagnoses arise regarding the potential relationship and pathogenesis of the tumor components, including hybrid tumor, collision tumor, and malignant transformation or high-grade transformation [2, 4, 10]. A hybrid salivary gland tumor has been defined as the coexistence of two distinct salivary gland tumor entities originating within the same topographical area. In contrast, a collision tumor refers to a meeting of two malignant neoplasms that arise at independent topographical sites but subsequently grow into and invade each other; this terminology has most frequently been used to describe tumors of distinct histogenesis (i.e., carcinoma and lymphoma) [4, 11]. Malignant transformation is the process whereby a benign neoplasm undergoes biologic transformation to a malignant neoplasm, most commonly manifested as carcinoma ex pleomorphic adenoma in the salivary glands. An analogous process is that of high-grade transformation (previously called dedifferentiation) in which a (usually lower grade) carcinoma with conventional morphology is associated with progression to a high-grade pleomorphic carcinoma, a change that often portends biologically aggressive behavior [12]. Significant overlap may exist within these concepts; indeed, a review of all 38 cases of salivary gland hybrid tumor published in the literature as of 2016 raised the argument that the majority of the previously diagnosed hybrid tumors may actually represent the high-grade transformation in a low-grade neoplasm, except for the few reported cases involving the coexistence two benign salivary gland tumor components [4].

The current case fulfills the definition a hybrid salivary gland tumor as it demonstrated two distinct, well-established salivary gland primary tumor histologies (MEC and BCA) presenting as a single primary tumor within one region of the parotid gland [5]. BCA has been found to be present with either adenoid cystic carcinoma or canalicular adenoma in hybrid tumors [2], but, to our knowledge, this is the first reported hybrid salivary gland tumor with the combination of MEC and BCA components. BCAs have been histologically classified into the solid type, tubular type, trabecular type, and membranous type [13]. To be noted, it is necessary to differentiate BCAs from intercalated duct lesions (IDLs), especially when the lesion is small such as in the current case and particularly with the tubular variant of BCA. This may be challenging because IDLs, ranging from hyperplasia to encapsulated adenoma architecturally, may have some morphologic overlap with BCAs [14, 15]. A recent review of 34 IDLs in 32 patients also showed that among other salivary gland tumors, IDLs most frequently coexisted with BCAs, raising the possibility of IDL being a precursor lesion to BCA [14]. Histologically, IDLs show proliferation of intercalated ducts, with frequent admixture with acinic cells and minimal intervening stroma. In the majority of IDLs, the individual ducts are composed of single layer duct cells with no readily discernable myoepithelial or basal cells. The ductal cells exhibit small, bland nuclei and eosinophilic or amphophilic cytoplasm [14, 15]. In our case, the lesion showed a well-circumscribed multinodular proliferation of typical basaloid cells, absence of acinic cells, and abundant eosinophilic hyalinized stroma surrounding the tumor cell nests in a characteristic ribbon-like pattern. Immunohistochemically, CK5/6 and p63 stains highlighted the prominent abluminal component. These histological and immunohistochemical features made the diagnosis of a membranous-type BCA more appropriate than that of an IDL [14].

The pathogenesis of hybrid tumors has not been clear in the literature, but it has been suggested that the two components share a single origin based on the frequent presence of a transitional morphology between the different tumor components [2, 4]. Our case, however, lacked a transition between the distinct tumor histologies, but instead showed a clear demarcation, a pattern frequently seen malignant transformation or high-grade transformation. MEC arising from a preexisting membranous-type BCA is certainly a reasonable explanation for the histologic findings. Indeed, the malignant transformation of BCA to nonbasaloid carcinomas has been reported, although extremely rare [10, 16]. Salivary duct carcinoma and adenocarcinoma, not otherwise specified, are the only nonbasaloid carcinomas arising from BCAs reported so far [10, 16]. In particular, the membranous type of BCA is believed to be pathogenetically distinct from the other forms of BCA. It has a higher recurrence rate reported as 25%, likely due to its tendency for multinodular growth, and is also more likely to undergo malignant transformation [17–22].

Molecular studies, thus far very limited in salivary gland hybrid tumors, may provide insight into their pathogenesis. To support the argument of malignant transformation of BCA in the current case, one would expect for the MEC and BCA components to share overlapping pathogenic genetic changes, leading to neoplasia (the formation of BCA) with subsequent gains of additional alterations leading to malignancy (the development of MEC). However, molecular analysis failed to specifically support a common origin for the two components in this case. While the tumor components shared 5 genetic changes (*CIC*,* RAF1, FGF3*,* PBRM1*, and* MCL1*), all were reported as VUS. Molecular guidelines recommend that continued efforts are undertaken to reclassify VUS variants as benign or pathogenic as more information becomes available, that labs continually search available clinical databases to further inform the potential significance of a VUS and that results are interpreted by a board-certified clinical molecular geneticist or molecular genetic pathologist or the equivalent (in this instance, one of the authors, LJT) [23]. Four of the five variants reported as VUS (*CIC*,* RAF1, FGF3*, and* MCL1*) have been previously reported in the Single Nucleotide Polymorphism database (dbSNP) (https://www.ncbi.nlm.nih.gov/snp, last accessed 3/5/2019) as germline single nucleotide polymorphisms (SNPs) (Table 1). The fifth variant,* PBRM1* L145F, is not reported in any of the databases interrogated (dbSNP, ClinVar, COSMIC) and is predicted to be benign by PolyPhen2 (http://genetics.bwh.harvard.edu/pph2/, last accessed 3/5/2019) [24–26]. The two additional variants reported in the MEC component (Table 1) are not reported in any of the databases interrogated and remain VUS although the possibility of a sequencing artifact cannot be excluded. Therefore, the overall evidence (or lack thereof in the sequence variants have not previously been reported in neoplasia) suggests that these changes are likely to represent germline variants [24–26]. The FoundationOne® assay tests tumor only without a germline sample control; therefore, germline variants may be classified as VUS.

Meanwhile, each tumor component harbored a potential driver genetic change:* BAP1* in MEC;* CREBBP* and* GNAS* in BCA. The* BAP1* (BRCA1-associated protein 1) protein has been found commonly altered in MEC [27]. It functions as a tumor suppressor and plays vital roles in transcription regulation and DNA damage repair. However, the exact role of* BAP1* in MEC oncogenesis needs further investigation. Alterations of* CREBBP* and* GNAS* genes have also been found to be involved in tumorigenesis of salivary gland malignancies such as MEC and adenoid cystic carcinoma but thus far not in BCA [27, 28]. Molecularly, BCA is frequently characterized by activating mutations in* CTNNB1* which result in nuclear accumulation of ß-catenin, a finding that can be detected immunohistochemistry. However, nuclear ß-catenin expression is uncommon in the membranous type of BCA, a subtype frequently characterized by* CYLD1* mutations and loss [29, 30]. The BCA component of our current case was negative for* CTNNB1 *alteration by NGS, and* CYLD1 *status is unknown as this gene is not covered in the NGS panel.

While we strongly favor independent derivation of MEC and BCA based on presently available molecular data, the possibility of malignant transformation cannot be entirely excluded. It is possible that the molecular testing failed to detect* GNAS* and* CREBBP* mutations in the MEC component, for instance, due to low allele frequency; allele frequencies are not provided in the FoundationOne® report. Alternatively, the neoplastic transformation might be due to a molecular change undetected by our analysis, potentially occurring at an early stage of development.

Assuming the MEC did not transform from the BCA, what potential factors might explain the coexistence of two unique salivary gland tumors in such intimate proximity? The term* field effect* has been used since 1953 to convey the concept of tissue predisposed to the development of neoplasia due to a field of cellular and molecular alterations. Recent reimaging of this concept has advocated for a broadening of potential factors to include environmental, genetic, dietary, lifestyle, microbial, and hormonal factors (*the exposome*) as well as their interactions (*the interactome*) [31]. In our patient's case, the history of tobacco use is one likely contribution to this field effect. Perhaps a local predisposition to neoplasia led to the simultaneous development of BCA and MEC; or perhaps predisposing factors more generally affected the patient's major salivary gland tissue given the presence of an ipsilateral salivary duct cyst and contralateral Warthin tumor. Alternatively, it is possible that the establishment of one tumor type altered the local tissue environment to locally predispose to the development of a different tumor type.

In summary, this case expands the spectrum of reported hybrid salivary gland tumors, documenting the novel coexistence of BCA and MEC. Molecular analysis raises additional questions about the purported pathogenesis of these rare neoplasms. In hybrid salivary gland tumors, the clinical history and imaging studies are nonspecific, and the diagnosis is made by histologic examination. Given that the relative proportion of the two tumor components may vary and treatment is based on the more aggressive component, thorough tumor sampling is necessary to render an accurate diagnosis and appropriate treatment strategy. In particular, adequate sampling at the time of intraoperative consultation is advisable so that a malignant component, if present, can be detected to guide surgical therapy towards total parotidectomy and lymph node sampling if appropriate, such as in the current case. Clinically, our patient is currently recovered uneventfully with no evidence of recurrence at four months from initial diagnosis.

## Figures and Tables

**Table 1:** Gene alterations in BCA and MEC components.

	BCA	MEC
Genomic alterations	*CREBBP* (Q355fs*∗*12)	
	*GNAS* (Q227L)	
		*BAP1* (S489fs*∗*82)

Variants of unknown significance	*CIC* (A851V) rs45596843	*CIC* (A851V) rs45596843
	*FGF3* (T79M) rs376992420	*FGF3* (T79M) rs376992420
	*MCL1* (M120V) rs151065075	*MCL1* (M120V) rs151065075
	*RAF1* (V312A) rs370243307 *PBRM1* (L145F)^∧^	*RAF1* (V312A) rs370243307 *PBRM1* (L145F)^∧^
		*EPHA7* (I808M)^∧∧^
		*FUBP1* (G25E)^∧∧^

Abbreviations: BCA, basal cell adenoma; MEC, mucoepidermoid carcinoma; rs, reference sequence. Symbols: ^∧^see text, predicted benign; ^∧∧^see text, possible sequencing artifacts.
